# OLDER ADULT FALLS AND LIMITATIONS IN ACTIVITIES OF DAILY LIVING

**DOI:** 10.1093/geroni/igae098.0230

**Published:** 2024-12-31

**Authors:** Yara Haddad, Ketra Rice, Jufu Chen, Ramakrishna Kakara

**Affiliations:** Centers for Disease Control and Prevention, Atlanta, Georgia, United States; Centers for Disease Control and Prevention, Atlanta, Georgia, United States; Centers for Disease Control and Prevention, Atlanta, Georgia, United States; Centers for Disease Control and Prevention, Atlanta, Georgia, United States

## Abstract

Falls among older adults (65+) often lead to injury and loss of independence. Our study aimed to estimate the odds of developing limitations in activities of daily living (ADL) and instrumental activities of daily living (IADL) after a fall. We used 2015-2020 Medicare Current Beneficiary Survey data to estimate the odds of developing ADL/IADL limitations in the year following a fall and examined risk and protective factors for developing them. Using logistic regression, we calculated crude and adjusted odds ratios (OR) and 95% Confidence Intervals (CI), adjusting for demographic, economic, comorbidity, and physical activity variables. Older adults reporting a fall had twice the odds of developing limitations in ADLs (OR=2.02; CI:1.70-2.39) or IADLs (OR=2.01; CI:1.72-2.34) the following year compared with those who did not fall. Among those who fell, adjusted ORs for developing ADL or IADL limitations were significantly higher for adults aged 75+ compared with those 65-74 but were significantly lower for adults who engaged in moderate or vigorous activity for ≥ 2 hours per week during baseline compared with those who did not. Although the likelihood of developing limitations after a fall may increase with age, results suggest engaging in moderate or vigorous physical activity of ≥ 2 hours per week may be protective. Regular physical activity with structured strength and balance exercises has been shown to reduce falls risk. The Stopping Elderly Accidents Deaths and Injuries (STEADI) initiative encourages health care providers to screen and assess for falls risk and intervene using strategies such as physical therapy.

